# Clinical Usefulness of the 2010 Clinical and Laboratory Standards Institute Revised Breakpoints for Cephalosporin Use in the Treatment of Bacteremia Caused by *Escherichia coli* or *Klebsiella* spp.

**DOI:** 10.1155/2015/831074

**Published:** 2015-02-22

**Authors:** Nam Su Ku, Hae-Sun Chung, Jun Yong Choi, Dongeun Yong, Kyungwon Lee, June Myung Kim, Yunsop Chong

**Affiliations:** ^1^Department of Internal Medicine and AIDS Research Institute, Yonsei University College of Medicine, Seoul, Republic of Korea; ^2^Department of Laboratory Medicine, Ewha Womans University School of Medicine, Seoul, Republic of Korea; ^3^Department of Laboratory Medicine and Research Institute of Bacterial Resistance, Yonsei University College of Medicine, Seoul, Republic of Korea

## Abstract

We investigated the clinical usefulness of the revised 2010 Clinical and Laboratory Standards Institute (CLSI) breakpoints for *Escherichia coli* and *Klebsiella* spp. Of 2,623 patients with bacteremia caused by *E. coli* or *Klebsiella* spp., 573 who had been treated appropriately with cephalosporin based on the CLSI 2009 guidelines were enrolled. There were no differences in the rates of treatment failure or mortality between the appropriately and inappropriately treated groups according to the CLSI 2010 guidelines. Additionally, in the matched case-control analysis, the treatment failure rate was higher in bacteremic patients with extended-spectrum *β*-lactamase- (ESBL-) producing but cephalosporin-susceptible organisms than in those with ESBL-nonproducing isolates when patients with urinary tract infections were excluded (44% and 0%, resp., *P* = 0.026). In patients with bacteremia caused by *E. coli* or *Klebsiella* spp., the revised CLSI 2010 guidelines did not lead to poorer outcomes. However, ESBL production appeared to be associated with poor clinical outcomes in patients with bacteremia from sources other than the urinary tract.

## 1. Introduction

In January 2010, revised cephalosporin breakpoints were published by the Clinical and Laboratory Standards Institute (CLSI) (Table 1) [1]. These new breakpoints were determined using the pharmacokinetic-pharmacodynamic properties of antimicrobial agents and minimal inhibitory concentration (MIC) distributions for relevant organisms [2, 3]. However, there were limited clinical data to support the efficacy of these new guidelines [4, 5]. On adhering to the CLSI 2010 guidelines, carbapenems will most likely be used as alternative antimicrobial agents owing to an increase in cephalosporin resistance, which will result in an increase in carbapenem administration [6]. Additionally, routine testing for extended-spectrum *β*-lactamase (ESBL) is no longer considered necessary before reporting susceptibility profiles that guide clinical management [1]. However, whether ESBL testing is important to ensure appropriate therapy [7] and to document local ESBL prevalence data, so that empiric therapy is as targeted as possible [8], is a matter of debate.

Therefore, we investigated the clinical usefulness of the revised breakpoints in the CLSI 2010 guidelines for the treatment of bacteremia caused by Enterobacteriaceae, specifically* Escherichia coli *and* Klebsiella *spp., compared with the CLSI 2009 guidelines.

## 2. Materials and Methods

### 2.1. Subjects and Study Design

**Figure 1:**
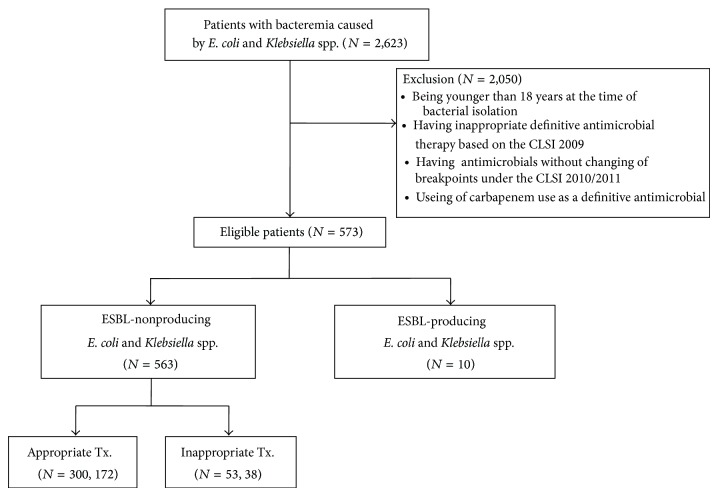
Flowchart of patient inclusion and subsequent treatment.

We conducted a retrospective cohort study to evaluate the clinical usefulness of the CLSI 2010 guidelines for the treatment of bacteremia caused by* E. coli *or* Klebsiella *spp. A medical record review was conducted for 2,623 patients who were treated for* E. coli *or* Klebsiella *spp. bacteremia between January 2006 and December 2010 at Severance Hospital, a tertiary-care teaching hospital with more than 2000 beds. The patients who were appropriately treated with extended-spectrum cephalosporins (ceftazidime, ceftriaxone, cefotaxime, or cefpiramide) were included and divided into 2 groups based on the appropriateness of antimicrobial therapy according to the CLSI 2010 guidelines (Figure 1). Clinical outcomes were compared between the 2 groups with respect to the class of antimicrobials used and the microbial species. Patients <18 years of age at the time of bacterial isolation and patients who received inappropriate definitive antimicrobial therapy based on the CLSI 2009 guidelines were excluded.

In addition, a matched case-control study was conducted to determine the clinical usefulness of ESBL testing. The clinical outcomes of sepsis patients with ESBL-producing cephalosporin-susceptible isolates who received a cephalosporin as the appropriate definitive antimicrobial treatment as per the CLSI 2010 guidelines were compared to matched subjects with sepsis caused by ESBL-nonproducing isolates (in ratio of 1 : 2). The 2 groups were matched for age, source of infection, and Sequential Organ Failure Assessment (SOFA) score [9].

The following variables identified from medical records and the computerized database in the clinical microbiology laboratory were assessed: age, gender, underlying disease, predisposing conditions, possible route of infection, laboratory data at the time of bacteremia onset and 72 hours after definitive antimicrobial therapy, results of antimicrobial susceptibility testing, antimicrobial regimen, severity of illness as determined by the SOFA score, and clinical outcome. The primary outcome measures were treatment failure and all-cause 28-day mortality rate. This study was approved by the Institutional Review Board (IRB) of Severance Hospital (IRB #4-2010-0522), and the need for written informed consent from all participants was waived by the approving IRB.

### 2.2. Definitions

Significant bacteremia was defined as isolation of* E. coli *or* Klebsiella *spp. in ≥1 separately obtained blood culture and the presence of clinical features compatible with fever and sepsis syndrome [10]. Hospital-acquired bacteremia was defined as a positive blood culture taken from a patient who demonstrated clinical evidence of infection ≥48 hours after admission [11]. The route of infection was determined based on isolation of the organism from the presumed entry point in conjunction with clinical evaluation [12]. Septic shock was defined as sepsis associated with evidence of organ hypoperfusion and either a systolic blood pressure of <90 mmHg or >30 mmHg less than baseline or required use of a vasopressor to maintain adequate blood pressure [13]. All underlying diseases, including cardiovascular disease, chronic renal disease, chronic liver disease, and chronic lung disease, were defined according to the International Classification of Disease, 10th Revision [14]. The Charlson index was used to assess the burden of chronic disease [15]. Definitive antimicrobial therapy was defined as antimicrobial therapy that began or continued on the day that the antibiogram results were reported to the clinicians, which was not later than 120 h after the initial positive blood sample had been drawn [16]. Susceptibility to cephalosporins was defined as* in vitro* susceptibility to cefotaxime or ceftazidime. Antimicrobial therapy was considered appropriate if the treatment regimen included antibiotics that were susceptible [16] and the dosage and route of antimicrobial administration were in accordance with current standards of care and their renal function [1, 17]. Treatment failure was defined as persistent fever, septic shock, or bacteremia 72 hours after starting definitive antimicrobial therapy [16]. Death was considered to be related to bacteremia if the patient died within 28 days after receiving treatment, unless clinical data clearly suggested that death was due to another cause.

### 2.3. Microbiological Tests

Isolates were identified using one of two conventional biochemical methods: ATB 32 GN or VITEK 2 systems (bioMerieux, Marcy-l'Etoile, France). Antimicrobial susceptibilities were determined using the disc-diffusion method or a VITEK-2 N131 card (bioMerieux, Hazelwood, MO, USA). The results were interpreted according to the CLSI 2009 and 2010 guidelines [1, 17]. ESBL production was determined using a double-disk potentiation test with amoxicillin-clavulanic acid and cefotaxime, ceftazidime, or cefepime or by positive results for ESBL on using the VITEK-2 N131 card.

### 2.4. Statistical Analysis

Student's* t*-tests were used to compare continuous variables, and *χ* ^2^ or Fisher's exact tests were used to compare categorical variables. All *P* values were two-sided, and *P* values < 0.05 were considered statistically significant. All statistical analyses were performed using SAS version 9.1.3 (SAS Institute, Cary, NC, USA).

## 3. Results

### 3.1. Baseline Characteristics and Microbiologic Data of the Subjects

Of the 2,623 patients with* E. coli *or* Klebsiella *spp. bacteremia, 573 patients were eligible for this study (Figure 1).  Table 2 shows the demographic and clinical characteristics of the patients. The median age was 65.5 years (range, 24–92 years), and 50.8% of the subjects were men. Of the 573 cases of bacteremia, 81.7% were community acquired, 18.3% were hospital acquired, 45.9% were associated with underlying malignancy, and 29.5% were associated with diabetes mellitus. The most frequent cause of sepsis was pancreatobiliary infection (46.6%), followed by urinary tract infection (UTI) (28.6%) and primary bacteremia (9.4%). The numbers of isolated* E. coli *and* Klebsiella *spp. were 360 (62.8%) and 213 (37.2%), respectively. Of these isolates, 1.7% (10/573) were ESBL producers.

### 3.2. Usefulness of the Revised Cephalosporin Breakpoints in the CLSI 2010 Guidelines for the Treatment of Bacteremia Caused by ESBL-Nonproducing* E. coli* and* Klebsiella *spp

A total of 353 and 210 cases of sepsis caused by ESBL-nonproducing* E. coli *and* Klebsiella *spp., respectively, were treated with a cephalosporin as appropriate definitive antimicrobial therapy according to the CLSI 2009 guidelines.  Table 3 shows the clinical characteristics and outcomes of these patients. Treatment with cephalosporin was considered to be inappropriate by the CLSI 2010 guidelines in 53 (53/353, 15.0%) and 38 (38/210, 18.1%) patients with identified* E. coli *and* Klebsiella *spp. isolates, respectively. Baseline characteristics did not differ between groups of appropriately treated and inappropriately treated patients, according to the 2010 guidelines. Treatment failure rates did not differ significantly between the appropriately and inappropriately treated groups as well (10.3% and 15.1% for* E. coli* (*P* = 0.308), resp.; 14.0% and 23.7% for* Klebsiella *spp. (*P* = 0.136), resp.). Additionally, there were no differences between the 2 groups in terms of 28-day mortality rates (2.7% and 7.5% for* E. coli *(*P* = 0.089), resp.; 8.7% and 2.6% for* Klebsiella *spp. (*P* = 0.315), resp.).

### 3.3. Matched Case-Control Study of Patients Treated with a Cephalosporin as an Appropriate Definitive Antimicrobial Treatment under the Revised CLSI Guidelines

In patients treated with a cephalosporin as appropriate definitive antimicrobial therapy according to the revised CLSI 2010 guidelines, a total of 10 patients with ESBL-producing isolates were identified (Table 4). There were no significant differences between the ESBL-producing and ESBL-nonproducing groups in baseline characteristics (Table 5). The treatment failure rate in the ESBL-producing group was 30.0% (3/10), while 10.0% (2/20) of the ESBL-nonproducing group failed in treatment (*P* = 0.300). Additionally, there were no significant differences between the ESBL-producing and -nonproducing groups in 28-day mortality rates (10% [1/10] versus 5% [1/20], *P* = 1.000).

We conducted a subgroup analysis of the matched case controls excluding patients with UTIs. The case group included 7 patients with ESBL-producing isolates that were matched according to age, origin of infection, and SOFA score with ESBL-nonproducing isolates (1 : 2 ratio) (Table 5). The treatment failure rates were 42.9% (3/7) and 0.0% (0/14) in the ESBL-producing and ESBL-nonproducing groups, respectively (*P* = 0.026). However, the 28-day mortality rates were not significantly different between the groups (14.3% [1/7] versus 7.1% [1/14], *P* = 1.000).

## 4. Discussion

In our study, the use of the revised CLSI 2010 guidelines did not lead to poorer clinical outcomes for patients treated with cephalosporins for* E. coli* and* Klebsiella* spp. bacteremia, compared with the CLSI 2009 guidelines. This is despite the fact that the revised breakpoints for extended-spectrum cephalosporins in the CLSI 2010 guidelines were likely to result in lower susceptibility rates and, in particular, routine testing for ESBL was no longer considered necessary, which led to our hypothesis that the revised guidelines would lead to poorer outcomes than the previous guidelines.

A recent study evaluating the effects of the clinical breakpoint changes in the revised CLSI 2010 guidelines on antibiotic susceptibility test reporting of gram-negative bacilli [6] reported no significant changes in the antibiotic susceptibility rates of* E. coli *or* Klebsiella *spp. to third-generation cephalosporins. Further, the majority of the changes that occurred were shifts from “susceptible” to “intermediate” susceptibility, indicating that, in fact, there may be little change in microbiologic susceptibility with the 2010 revised breakpoints.

Despite a lack of statistical significance in the present study, there were potentially clinically significant changes in outcomes. For example, in patients with bacteremia caused by ESBL-nonproducing* E. coli* and* Klebsiella* spp. treated with a cephalosporin according to the new CLSI breakpoints, the 28-day mortality rates, based on the appropriateness of treatment, were 2.7% (appropriate) and 7.5% (inappropriate) for* E. coli* and 8.7% (appropriate) and 2.6% (inappropriate) for* Klebsiella *spp. However, these results may have been influenced by fewer occurrences at the endpoints. Additionally, in regards to treatment failure, if the whole series is considered, a higher proportion of patients inappropriately treated failed (18.6% [17/91] versus 11.6% [55/472], *P* = 0.07). Thus, even if there was no statistically significant association between appropriateness of therapy and clinical failure, there was a trend towards significance between them.

The revised CLSI guidelines suggest that, with the new breakpoints, routine ESBL testing is no longer necessary before reporting results that will guide clinical management [1]. In this study, we also found that, in patients treated with a cephalosporin as the appropriate definitive antimicrobial therapy under the revised CLSI 2010 guidelines, ESBL production did not influence clinical outcomes in patients with* E. coli *and* Klebsiella *spp. bacteremia. However, many investigators still debate whether ESBL testing is important to increase the probability of success [7]. In addition, when we excluded patients with UTIs as the source of bacteremia, the treatment failure rate was significantly higher in the group with bacteremia due to ESBL-producing isolates than in the group with bacteremia due to ESBL-nonproducing isolates. Thus, our data suggest that ESBL testing may be considered for patients with bacteremia from sources other than UTI. This supports previous reports that have shown that patients with susceptible MICs and ESBL-producing isolates frequently experienced antimicrobial treatment failure [5, 18, 19] and that, in bacteremic patients with ESBL-producing isolates, UTI was an independent determinant of reduced mortality rates [20].

Our study has certain limitations. First, the sample of patients with* E. coli *and* Klebsiella *spp. bacteremia in this study was collected from a single center; this may limit the generalizability of the results to other centers. Second, as in all retrospective studies, there is potential for bias and inaccurate data collection. Third, despite a large initial study sample, subanalysis was conducted in a small sample of patients. Further prospective studies conducted in multiple centers with larger samples are necessary to appraise the clinical usefulness of the revised CLSI breakpoints. In addition, the appropriateness of the initial empirical antimicrobials could have had an influence on the clinical outcomes of this study. However, because 1.2% (6/482) of the appropriate definitive antimicrobials had inappropriate empirical antimicrobials and only 3.3% (3/91) of the inappropriate definitive antimicrobials had appropriate empirical antimicrobials, we think that the initial empirical antimicrobials were likely to have little influence on the clinical outcomes in this study. Lastly, in our hospital, antimicrobial susceptibilities were determined using the disc-diffusion method only until May 2009, and since then, MICs were measured and reported by using VITEK-2 system. Thus, we were not able to describe the distribution of the MICs of organisms.

## 5. Conclusions

In conclusion, in patients with bacteremia caused by* E. coli *or* Klebsiella *spp., treatment according to the revised CLSI 2010 guideline did not lead to poorer outcomes, compared to treatment according to the CLSI 2009 guideline. However, ESBL production appeared to be associated with poor clinical outcomes in patients with bacteremia from sources other than the urinary tract.

## Figures and Tables

**Table 1:** Minimum inhibitory concentration and disk diffusion interpretive breakpoints for several cephalosporins established in January 2009 and January 2010 by the Clinical and Laboratory Standards Institute.

Agent	MIC breakpoints (*μ*g/mL)						Disk diffusion breakpoints (mm)					
	CLSI 2009 (M100-S19)			CLSI 2010 (M100-S20)			CLSI 2009 (M100-S19)			CLSI 2010 (M100-S20)		
	S	I	R	S	I	R	S	I	R	S	I	R
Cefazolin	≤8	16	≥32	≤1	2	≥4	≥18	15–17	≤14	NA	NA	NA
Cefotaxime	≤8	16–32	≥64	≤1	2	≥4	≥23	15–22	≤14	≥26	23–25	≤22
Ceftizoxime	≤8	16–32	≥64	≤1	2	≥4	≥20	15–19	≤14	≥25	22–24	≤21
Ceftriaxone	≤8	16–32	≥64	≤1	2	≥4	≥21	14–20	≤13	≥23	20–22	≤19
Ceftazidime	≤8	16	≥32	≤4	8	≥16	≥18	15–17	≤14	≥21	18–20	≤17

MIC, minimum inhibitory concentration; CLSI, Clinical and Laboratory Standards Institute; NA, not available.

**Table 2:** Demographic and clinical characteristics of 573 patients with *E. coli *or *Klebsiella *spp. Bacteremia.

Variables	Number (%)
Mean age (range, years)	65.51 (24–92)
Male	291 (50.8)
Hospital acquired	105 (18.3)
Community acquired	468 (81.7)
Underlying disease	
Malignancy	
Solid	249 (43.5)
Hematologic	14 (2.4)
Transplantation	
Solid	5 (0.9)
Hematopoietic stem cell	0 (0.0)
Diabetes	169 (29.5)
Cardiovascular disease	261 (45.5)
Chronic lung disease	4 (0.7)
Chronic renal disease	23 (4.0)
Chronic liver disease	58 (10.1)
HIV infection	1 (0.1)
Route of infection	
Urinary tract infection	164 (28.6)
Liver abscess	40 (7.0)
Pancreatobiliary infection	267 (46.6)
Pulmonary infection	17 (3.0)
Peritonitis	18 (3.1)
Primary bacteremia	54 (9.4)
Other	13 (2.3)
Charlson index, mean ± SD	2.63 ± 2.278
SOFA score, mean ± SD	3.02 ± 2.493
ESBL-producing isolates	10 (1.7)
28-day mortality	29 (5.1)

ESBL, extended-spectrum *β*-lactamase; HIV, human immunodeficiency syndrome; ICU, intensive care unit; SOFA, Sequential Organ Failure Assessment.

**Table 3:** Clinical characteristics and outcomes of patients with bacteremia caused by ESBL-nonproducing Enterobacteriaceaetreated with a cephalosporin according to the new CLSI breakpoints.

Variables	Number (%) of patients with bacteremia			Number (%) of patients with bacteremia		
	caused by *E. coli*:			caused by *Klebsiella *spp.:		
	Appropriate Tx.	Inappropriate Tx.	*P* value	Appropriate Tx.	Inappropriate Tx.	*P* value
	(*N* = 300)	(*N* = 53)		(*N* = 172)	(*N* = 38)	
Age (years), mean ± SD	66.06 ± 12.81	63.58 ± 13.59	0.199	65.21 ± 11.72	65.87 ± 11.72	0.753
Male	122 (40.7)	24 (45.3)	0.529	104 (60.5)	29 (76.3)	0.032
Hospital acquired	43 (14.3)	9 (17.0)	0.616	43 (25.0)	5 (13.2)	0.116
Community acquired	257 (85.7)	44 (83.0)	0.616	129 (75.0)	33 (86.8)	0.116
Underlying disease						
Malignancy						
Solid	120 (40.0)	22 (41.5)	0.836	89 (51.7)	14 (36.8)	0.096
Hematologic	7 (2.3)	1 (1.9)	1.000	3 (1.7)	1 (2.6)	0.717
Transplantation						
Solid	4 (1.3)	1 (1.9)	0.558	0 (0.0)	0 (0.0)	
Hematopoietic stem cell	0 (0.0)	0 (0.0)		0 (0.0)	0 (0.0)	
Diabetes	90 (30.0)	11 (20.8)	0.170	56 (32.6)	10 (26.3)	0.453
Cardiovascular disease	147 (49.0)	26 (49.1)	0.994	69 (40.1)	16 (42.1)	0.821
Chronic lung disease	1 (0.3)	0 (0.0)	1.000	3 (1.7)	0 (0.0)	1.000
Chronic renal disease	11 (3.7)	1 (1.9)	0.701	5 (2.9)	3 (7.9)	0.146
Chronic liver disease	28 (9.3)	4 (7.5)	0.800	24 (14.0)	2 (5.3)	0.179
HIV infection	1 (0.3)	0 (0.0)	1.000	0 (0.0)	0 (0.0)	
Charlson index	2.53 ± 2.24	2.28 ± 1.98	0.446	2.97 ± 2.47	2.21 ± 2.04	0.078
Source of infection						
Urinary tract infection	118 (39.3)	21 (39.6)	0.968	15 (8.7)	6 (15.8)	0.189
Liver abscess	7 (2.3)	1 (1.9)	1.000	26 (15.1)	7 (18.4)	0.612
Pancreatobiliary infection	132 (44.0)	24 (45.3)	0.862	88 (51.2)	18 (47.4)	0.672
Pulmonary infection	4 (1.3)	1 (1.9)	0.559	8 (4.7)	4 (10.5)	0.236
Peritonitis	7 (2.3)	2 (3.8)	0.629	9 (5.2)	0 (0.0)	0.369
Primary bacteremia	26 (8.7)	3 (5.7)	0.595	20 (11.6)	3 (7.9)	0.774
Others	6 (2.0)	1 (1.9)		4 (2.3)	0 (0.0)	
SOFA score, mean ± SD	2.75 ± 2.316	2.79 ± 2.315	0.910	3.39 ± 2.737	3.47 ± 2.275	0.860
Antimicrobials						
Ceftazidime	7 (2.3)	0 (0.0)	0.600	2 (1.2)	1 (2.6)	0.452
Cefpiramide	104 (34.7)	17 (32.1)	0.806	72 (41.9)	11 (28.9)	0.141
Cefotaxime	18 (6.0)	4 (7.5)	0.756	16 (9.3)	5 (13.2)	0.549
Ceftriaxone	171 (57.0)	32 (60.4)	0.660	82 (47.7)	21 (55.3)	0.397
Clinical outcomes						
Treatment failure after 72 hr	31 (10.3)	8 (15.1)	0.308	24 (14.0)	9 (23.7)	0.136
28-day mortality	8 (2.7)	4 (7.5)	0.089	15 (8.7)	1 (2.6)	0.315

CLSI, Clinical and Laboratory Standards Institute; ESBL, extended-spectrum beta lactamase; *E. coli*, *Escherichia coli*; HIV, human immunodeficiency syndrome; ICU, intensive care unit; SOFA, Sequential Organ Failure Assessment; SD, standard deviation; Tx., treatment.

**Table 4:** Clinical manifestations of 10 patients with a cephalosporin as an appropriate definitive antimicrobial treatment under revised CLSI 2010 guidelines.

Number	Gender/age	Organisms	Site of acquisition	Underlying disease	Site of infection	Septic shock at the time of bacteremia	Definitive antimicrobials	CLSI 2010	ESBL-producing	Treatment failure^a^	28-day mortality
1	M/45	KOX	HA	AGC	Biliary	Yes	Ceftriaxone^b^	Susceptible	Yes	Yes	Yes
2	F/46	KPN	HA	ESRD on HD	Primary	No	Ceftriaxone^b^	Susceptible	Yes	No	No
3	M/56	ECO	CA	AGC	Biliary	Yes	Cefpiramide^c^	Susceptible	Yes	No	No
4	M/63	ECO	CA	CBD stone, CKD, megaloblastic anemia	Biliary	No	Cefpiramide^c^	Susceptible	Yes	Yes	No
5	M/64	ECO	HA	Mantle cell lymphoma	Primary	No	Ceftriaxone^b^	Susceptible	Yes	No	No
6	M/64	KOX	HA	Rectal cancer	Urinary tract	No	Cefpiramide^c^	Susceptible	Yes	No	No
7	M/67	ECO	HA	HCC (B-viral)	Biliary	Yes	Cefotaxime^d^	Susceptible	Yes	Yes	No
8	F/72	ECO	CA	DM, CKD	Urinary tract	No	Ceftriaxone^b^	Susceptible	Yes	No	No
9	M/81	ECO	CA	CBD stone	Biliary	Yes	Cefpiramide^c^	Susceptible	Yes	No	No
10	F/84	ECO	CA	DM	Urinary tract	No	Ceftriaxone^b^	Susceptible	Yes	No	No

CLSI, Clinical and Laboratory Standards Institute; ESBL, extended-spectrum *β*-lactamase; ECO, *Escherichia coli*; KPN, *Klebsiella pneumonia*; KOX, *Klebsiellaoxytoca*; HA, hospital acquired; CA, community acquired; AGC, advanced gastric cancer; ESRD, end stage renal disease; HD, hemodialysis; CBD, common bile duct; HCC, hepatocellular carcinoma; DM, diabetes mellitus; CKD, chronic kidney disease.

^a^72 hrs after administration of definitive antimicrobials.

^b^2.0 g intravenous once a day.

^c^1.0 g intravenous three times a day.

^d^1.0 g intravenous three times a day.

**Table 5:** Clinical characteristics and outcomes of patients treated with a cephalosporin as an appropriate definitive antimicrobial treatment under the revised CLSI guidelines in ESBL-producing or nonproducing *E. coli *and *Klebsiella *spp. bacteremia.

Variables	Patients with bacteremia caused by			Non-UTI Patients with bacteremia caused by		
	*Enterobacteriacea*e			*Enterobacteriacea*e		
	ESBL-producing	Non-ESBL-producing	*P* value	ESBL-producing	Non-ESBL-producing	*P*-value
	*N* = 10, *n* (%)	*N* = 20, *n* (%)		*N* = 7, *n* (%)	*N* = 14, *n* (%)	
Age (years), mean ± SD	64.261 ± 12.976	65.15 ± 11.47	0.849	60.34 ± 12.603	63.70 ± 10.265	0.519
Male	7 (70.00)	12 (60.00)	0.702	6 (85.7)	10 (71.4)	0.624
Organism						
*E. coli *	7 (70.00)	14 (70.00)	1.000	5 (71.4)	8 (57.1)	0.192
*K. pneumonia *	1 (10.00)	6 (30.00)	0.372	1 (14.3)	6 (42.9)	
*K. oxytoca *	2 (20.00)	0 (0.00)	0.103	1 (14.3)	0 (0.00)	
Hospital acquired	5 (50.00)	7 (35.00)	0.461	4 (57.1)	5 (35.7)	0.397
Community acquired	5 (50.00)	13 (65.00)		3 (42.9)	9 (64.3)	
Underlying disease						
Malignancy						
Solid	5 (50.00)	10 (50.00)	1.000	4 (57.1)	9 (64.3)	1.000
Hematologic	2 (20.00)	1 (5.00)	0.251	2 (28.6)	1 (7.1)	0.247
Transplantation						
Solid	0 (0.00)	2 (10.00)	0.540	0 (0.0)	2 (14.3)	0.533
Hematopoietic stem cell	0 (0.00)	0 (0.00)		0 (0.0)	0 (0.0)	
Diabetes	2 (20.00)	7 (35.00)	0.675	0 (0.0)	5 (35.7)	0.123
Cardiovascular disease	2 (20.00)	8 (40.00)	0.420	0 (0.0)	4 (28.6)	0.255
Chronic lung disease	0 (0.00)	1 (5.00)	1.000	0 (0.00)	1 (7.1)	1.000
Chronic renal disease	2 (20.00)	1 (5.00)	0.251	1 (14.3)	1 (7.1)	1.000
Chronic liver disease	0 (0.00)	1 (5.00)	1.000	0 (0.00)	1 (7.1)	1.000
Source of infection						
Urinary tract infection	3 (30.00)	6 (30.00)	1.000			
Pancreatobiliary infection	5 (50.00)	10 (50.00)	1.000	5 (71.4)	10 (71.4)	1.000
Primary bacteremia	2 (20.00)	4 (20.00)	1.000	2 (28.6)	4 (28.6)	1.000
SOFA score, mean ± SD	4.300 ± 3.653	4.300 ± 3.556	1.000	5.71 ± 3.450	5.71 ± 3.315	1.000
Antimicrobials						
Cefpiramide	4 (40.0)	5 (25.0)	1.000	3 (42.9)	5 (35.7)	1.000
Cefotaxime	1 (10.0)	3 (15.0)	1.000	1 (14.3)	2 (14.3)	1.000
Ceftriaxone	5 (50.0)	12 (60.0)	1.000	3 (42.9)	7 (50.0)	1.000
Clinical outcomes						
Treatment failure after 72 hr	3 (30.00)	2 (10.00)	0.300	3 (42.9)	0 (0.0)	0.026
28-day mortality	1 (10.00)	1 (5.00)	1.000	1 (14.3)	1 (7.1)	1.000

CLSI, Clinical and Laboratory Standards Institute; ESBL, extended-spectrum beta lactamase; *E. coli*, *Escherichia coli*; HIV, human immunodeficiency syndrome; ICU, intensive care unit; SOFA, Sequential Organ Failure Assessment; SD, standard deviation; Tx., treatment.
